# Supplementary material for: EnzML: multi-label prediction of enzyme classes using InterPro signatures
Source: BMC Bioinformatics. 2012 Apr 25;13:61. doi: 10.1186/1471-2105-13-61 (PMC3483700; doi:10.1186/1471-2105-13-61)
Supplement: Addtional file 5 — The Java code to format the data files, evaluate and predict. The file enzml_java_code.tar.gz contains the Java code used to format database data to ARFF and XML formats, to execute cross and train-test (jackknife) evaluations and to record evaluation results to database. More information is included in the readme.txt file and the Javadoc files. The code can be used with a MySQL database. To use a different database software, other JDBC drivers might be required. [file 1471-2105-13-61-S5.gz › java_code/enzml2011/doc/uk/ac/ed/inf/enzml/mulan/learn/package-summary.html]

uk.ac.ed.inf.enzml.mulan.learn


---


|  |  |  |
| --- | --- | --- |
| **Overview** \| **Package** \| Class \| **Use** \| **Tree** \| **Deprecated** \| **Index** \| **Help** | |  |
| **PREV PACKAGE**   **NEXT PACKAGE** | **FRAMES**    **NO FRAMES**     **All Classes** |  |


---

## Package uk.ac.ed.inf.enzml.mulan.learn

| **Class Summary** | |
| --- | --- |
| **EvaluationParameters** | Contains the evaluation parameters (micro-macro precision, micro-macro accuracy etc.) of a Multi label Mulan experiment. |
| **EvaluatorNoAUC** | Prepares the evaluation measures taking into consideration the dataset size. |
| **ExperimentTable** | Gives the names and table field names for multi-label specific evaluation measures (such as micro-accuracy, macro-accuracy, hamming loss etc.) |
| **MulanCrossEvaluator** | Class created to execute cross-evaluations and extract individual evaluation results (not possible from the Mulan class Evaluator, because only averaged results are returned). |
| **MulanCrossExperimenter** | Using Mulan (multi-label machine learning), this class initialises and executes either Train-Test evaluation (if two different arff records are given) (calling `MulanTrainTestEvaluator`) or cross-evaluations (calling `MulanCrossEvaluator`) if only one arff record is given. |
| **MulanLearner** | Containing a Mulan learner and its name, that is: the name of the MultiLabelLearnerBase plus the name of the algorithm within |
| **MulanLearners** | Class |
| **MulanSerializer** | Takes an arff file and an algorithm. |
| **ResultsFormatter** | Class |
| **ResultsSaver** | Outputs cross-evaluation results to screen, file and database. |

---


|  |  |  |
| --- | --- | --- |
| **Overview** \| **Package** \| Class \| **Use** \| **Tree** \| **Deprecated** \| **Index** \| **Help** | |  |
| **PREV PACKAGE**   **NEXT PACKAGE** | **FRAMES**    **NO FRAMES**     **All Classes** |  |


---
